# The Association of Real-World CA 19-9 Level Monitoring Patterns and Clinical Outcomes Among Patients With Metastatic Pancreatic Ductal Adenocarcinoma 

**DOI:** 10.3389/fonc.2021.754687

**Published:** 2021-10-04

**Authors:** Ben George, Matthew Kent, Andy Surinach, Neil Lamarre, Paul Cockrum

**Affiliations:** ^1^ Department of Medical Oncology, Froedtert & the Medical College of Wisconsin, Milwaukee, WI, United States; ^2^ Real World Data Analytics, Genesis Research, Hoboken, NJ, United States; ^3^ Oncology HEOR, Ipsen, Cambridge, MA, United States

**Keywords:** CA 19-9, metastatic pancreatic cancer, overall survival, prognostic factor, chemotherapy

## Abstract

**Background:**

Pancreatic cancer is expected to be the third deadliest cancer in the US in 2021. Evaluation of treatment response in patients with mPDAC necessitates scheduled clinical and radiographic assessments along with monitoring serum CA 19-9 levels. Currently available single-institution data examining the importance of CA 19-9 monitoring cannot be generalized to real-world settings. We investigated the impact of serum CA 19-9 monitoring and its association with clinical outcomes in patients with mPDAC in a population-based setting.

**Methods:**

Data were extracted from the Flatiron Health electronic health record (EHR)-derived de-identified database for patients diagnosed with mPDAC between January 1, 2015, and June 30, 2020. Serum CA 19-9 levels at baseline – defined as the values obtained ≤ 60 days prior to treatment initiation - and during treatment were extracted. CA 19-9 levels > 40 IU/mL were considered elevated. Survival outcomes were compared based on testing frequency, baseline CA 19-9 levels, and change in CA 19-9.

**Results:**

6,118 patients with mPDAC who received treatment were included in the analysis. The median age at diagnosis was 68 years (IQR: 61-75). Patients with normal baseline CA 19-9 experienced longer median survival than patients with elevated levels [1L: 8.8 months (95% CI: 7.9 - 10) *vs.* 7.2 months (6.8 – 7.5), p < 0.001; 2L: 7.2 months (6.1 – 9.2) *vs.* 5.2 months (4.9 – 5.6), p < 0.001; 3L: 6.1 months (5.4 – 9.1) *vs.* 3.9 months (3.4 – 4.3), p < 0.001]. Patients with decreasing/stable CA 19-9 during treatment experienced longer survival than patients who experienced an increase in CA 19-9 levels [1L: 10.9 months (10.5 – 11.3) *vs.* 5.4 months (5.1 – 5.9), p < 0.0001; 2L: 8.2 months (7.7 – 8.5) *vs.* 4.3 months (4.1 – 4.7), p < 0.001; 3L: 7.5 months (6.6 – 9.2) *vs.* 3.7 months (3.4 – 4.3), p < 0.001].

**Conclusions:**

In one of the largest, contemporary, real-world studies of patients with mPDAC, elevated CA 19-9 level at treatment initiation demonstrated a prognostic impact. Routine serial monitoring of CA 19-9 levels during treatment may be warranted, in addition to clinical and radiographic assessment, and may translate into better patient outcomes. Further validation studies are needed to understand the generalizability of these results.

## Introduction

Pancreatic cancer is a lethal malignancy and has the highest mortality rate among all cancers (1). Although pancreatic cancer accounts for an estimated 3.6% of all cancers in the US, it is currently the 3rd leading cause of cancer-related death in the US after lung and colon cancers (1). Pancreatic ductal adenocarcinoma (PDAC) is the most frequent type of pancreatic cancer, representing approximately 85% of cases (2). Over 48,220 deaths by PDAC are reported annually in the US, and the incidence rate of PDAC is rising year-over-year. Despite the rapid advancement of treatment options for PDAC patients in recent years, the survival rates remain abysmal (3). The 5-year relative survival rate for all patients diagnosed with PDAC is 10% while the survival rate for patients with metastatic disease is below 3% (1).

Surgery remains the only potentially curative treatment for patients with PDAC (4) Due to the propensity of PDAC cells to metastasize early, up to 80% of patients receive a diagnosis at an advanced stage, by which time the tumor is unresectable (4). Only 10-20% of patients would have resectable tumors after careful neoadjuvant treatment, and chemotherapy is the only option for metastatic patients (5, 6).

Due to the aggressive nature of the disease, regular monitoring of patients on PDAC treatment is performed using clinical assessments supplemented with radiographic imaging in order to determine response to treatment and rule out disease progression (7). Currently, serum carbohydrate antigen 19-9 (CA 19-9) is the only biomarker approved by the Food and Drug Administration (FDA) for the management of pancreatic cancer (8). Serum CA 19-9, a sialylated Lewis blood group antigen, is an antigen associated with pancreatic cancer (9). The sensitivity and specificity of CA 19-9 tests are 80% and 80-90% respectively (10). CA 19-9 has been validated as an effective prognostic biomarker that can be used to aid in treatment decisions for patients with metastatic PDAC (mPDAC) (11–16). The objective of this study was, for the first time in a large, contemporary database, to assess the real-world use and outcomes associated with serum CA 19-9 monitoring in a population-based setting of mPDAC patients.

## Methods

### Data Source

his retrospective descriptive analysis utilized the nationwide Flatiron Health^®^ longitudinal database, a demographically and geographically diverse database derived from electronic health record (EHR) data. This database includes data from over 280 cancer clinics representing approximately 800 sites of care and more than 2.4 million active US cancer patients. The majority of patients in the database originate from the community oncology setting. The database meets the requirements of the Health Insurance Portability and Accountability Act of 1996 for fully de-identified data sets and subject to obligations to prevent re-identification to protect patient confidentiality. As the study was observational in nature and utilizes de-identified patient data, it is exempt from institutional review board review.

### Study Population

For the primary study analysis, four mutually exclusive cohorts were constructed based on the CA 19-9 testing patterns. Patients were required to have an mPDAC diagnosis between January 1, 2015, and June 30, 2020 to be screened for eligibility. The date of the first-line (1L) treatment initiation during this timeframe denotes the study index date. Eligible patients were required to have at least 1 recorded activity within 90 days, on or after, their mPDAC diagnosis date. Patients were also required to be at least 18 years old at diagnosis, treated with 1L systemic therapy for PDAC, and have at least one recorded follow-up activity after the start of 1L treatment. If the patients were treated in second- (2L) and third-line (3L), they were required to have follow-up activity recorded in the database after initiating those respective lines of therapy. Exclusion criteria included the following: 1. absence of activity (visit/administration) on or after the respective index date; and 2. initiation of 1L therapy after the date of death.

### Study Measures

The primary study measure was CA 19-9 testing pattern during systemic treatment stratified by the timing of testing and the number of tests that occurred during treatment. Overall survival (OS) was characterized by CA 19-9 testing patterns. CA 19-9 test timing, the duration of treatment stratified by CA 19-9 testing, the proportion of patients who proceeded to the next line of therapy, and OS from treatment initiation were evaluated. OS was assessed from the start of each line of therapy and patients with a death event were assigned the 15^th^ day of the month of death as the event date. Patients without a death recorded in the database were censored at their last recorded clinical activity. OS was stratified based on the following: 1. CA 19-9 testing patterns (no tests observed, baseline tests, one test during treatment, multiple tests during treatment); 2. baseline CA 19-9 levels (Normal, Elevated, Missing); and 3. CA 19-9 change from baseline relative to the lowest value recorded during treatment (Decreasing/Same, Increasing, Missing).

Demographic characteristics, including age, gender, US geographic region (Northeast, South, Midwest, West, Other), race/ethnicity (Black, White, Hispanic, Asian, Other/Unknown), index year and practice type were assessed at the index date. Baseline clinical characteristics including stage at initial PDAC diagnosis, site of the primary tumor, Eastern Cooperative Oncology Group (ECOG) Performance Status (closest score within 30 days prior or 7 days after the start of systemic treatment), presence of any previous surgery, surgery type, and the baseline serum CA 19-9 levels within 60 days prior the start of therapy were assessed. CA 19-9 levels greater than 40 U/mL were considered elevated.

### Statistical Analysis

Descriptive analyses were performed for all study variables. Summary statistics such as mean and standard deviation were calculated for all continuous variables, and frequency counts and percentages were calculated for categorical variables. Kaplan-Meier methods were used to calculate median overall survival, and a p-value < 0.05 was considered statistically significant. Statistical significance for overall survival was evaluated using the log-rank test. All analyses were conducted using R (version 4.0.0).

## Results

### Study Cohort

8,776 patients were identified with mPDAC diagnosis between January 1, 2015, and June 30, 2020. Most (n=8,134) of these patients had recorded activity within 90 days, on or after, the metastatic diagnosis date and were > 18 years of age at mPDAC diagnosis. About three-quarters (n=6,142) of these patients were treated with 1L systemic therapy. 6,118 patients met all the study selection criteria (**Supplementary Figure 1**). The final study analysis included four mutually exclusive cohorts defined by CA 19-9 testing patterns: no testing cohort (n=781), baseline test only cohort (n=1,082), one test during 1L treatment cohort (n=896), and multiple tests during 1L treatment cohort (n=3,359).

### Demographic and Clinical Characteristics

Demographic and clinical characteristics for each treated cohort are presented in **Table 1**. Of the 6,118 mPDAC patients included, 39.3% (n=2,402) of patients were treated in 2L, and 12.9% (n=790) in 3L. The median age at metastatic diagnosis of patients across all cohorts was 68 years (IQR: 61-75), and 55% of the mPDAC patients were male. Overall, the majority of patients (67.1%) were White, while 8.5% were Black, 1.8% were Asian and only 0.2% were Hispanic. While all patients in the study were diagnosed with mPDAC, the majority (67.1%) of patients were initially diagnosed with stage IV disease while the remainder progressed to metastatic disease from earlier stage PDAC. Among patients who had their CA 19-9 assessed in the baseline period, most (84.7%) had elevated baseline CA 19-9.

**Table 1:** Patient demographics.

Characteristic	First Line Treated Patients, N = 6,118	Second Line Treated Patients, N = 2,402	Third Line Treated Patients, N = 790
**Sex**			
Female	2,782 (45%)	1,106 (46%)	372 (47%)
Male	3,336 (55%)	1,296 (54%)	418 (53%)
**Age**			
Mean [SD]	68 [10]	66 [10]	66 [9]
Median [IQR]	68 [61 - 75]	67 [60 - 73]	67 [60 - 73]
**Race**			
Asian	108 (1.8%)	54 (2.2%)	17 (2.2%)
Black or African American	519 (8.5%)	191 (8.0%)	53 (6.7%)
Hispanic or Latino	14 (0.2%)	6 (0.2%)	2 (0.3%)
White	4,106 (67%)	1,673 (70%)	582 (74%)
Other Race	788 (13%)	284 (12%)	82 (10%)
Missing/Unknown	583 (9.5%)	194 (8.1%)	54 (6.8%)
**Geographic Region**			
Midwest	698 (11%)	308 (13%)	100 (13%)
Northeast	913 (15%)	339 (14%)	104 (13%)
South	2,653 (43%)	990 (41%)	319 (40%)
West	847 (14%)	335 (14%)	110 (14%)
Unknown	1007 (16%)	430 (18%)	157 (20%)
**Stage IV at Initial Diagnosis**	4,120 (67%)	1,590 (66%)	540 (68%)
**Tumor Location**			
Body	1,188 (19%)	504 (21%)	169 (21%)
Head	3,045 (50%)	1,184 (49%)	385 (49%)
Overlapping Sites	588 (9.6%)	225 (9.4%)	72 (9.1%)
Pancreas, Nos	188 (3.1%)	61 (2.5%)	20 (2.5%)
Tail	1,109 (18%)	428 (18%)	144 (18%)
**ECOG PS**			
0	1,368 (22%)	473 (20%)	146 (18%)
1	2,045 (33%)	893 (37%)	315 (40%)
2+	795 (13%)	368 (15%)	119 (15%)
Missing	1,910 (31%)	668 (28%)	210 (27%)
**Progressed to next line**	2,324 (38%)	792 (33%)	217 (27%)
**Duration of therapy, weeks**			
Mean [SD]	17 [21]	14 [19]	17 [18]
Median [IQR]	10 [3 - 22]	8 [3 - 18]	12 [6 - 23]

ECOG, Eastern Cooperative Oncology Group; SD, Standard Deviation; IQR, Interquartile range.

### Testing and Treatment Patterns

**Figure 1:**
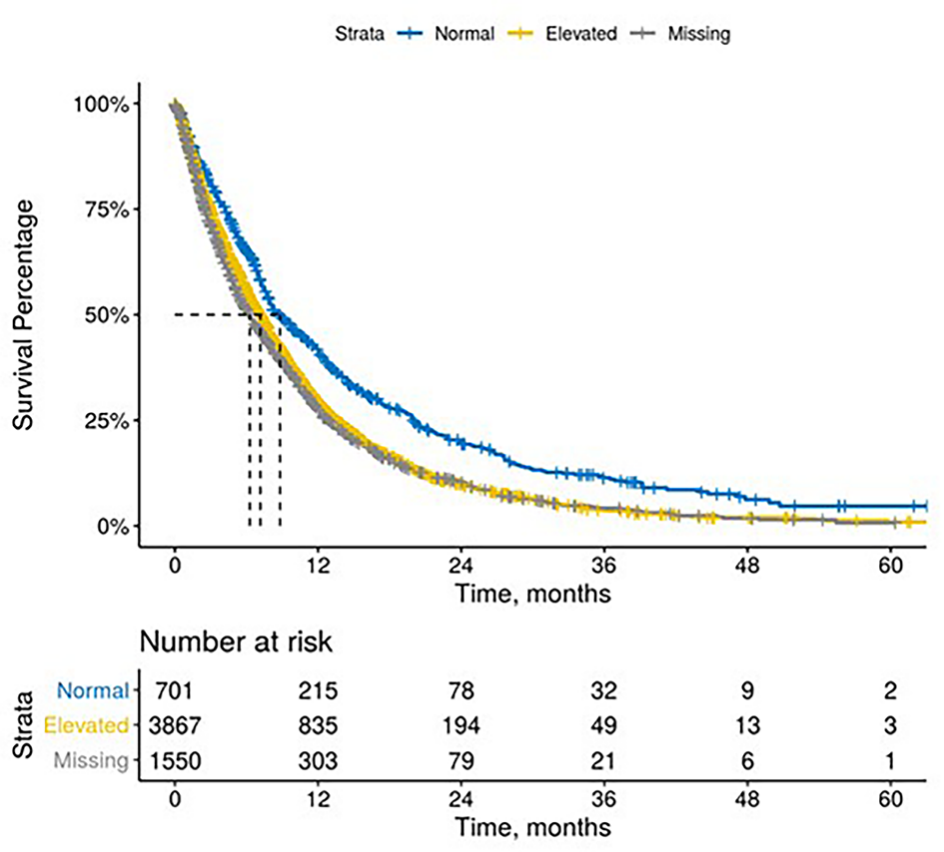
Overall survival by Baseline Carbohydrate Antigen 19-9.

The median time between CA 19-9 tests was 3.5 weeks (IQR 2.1 - 5.6). 63% of patients with elevated baseline CA 19-9 received multiple tests while 54% of patients with normal baseline CA 19-9 level received multiple tests (**Table 2**). The testing interval among patients with elevated baseline CA 19-9 was lower than patients with normal CA 19-9 level (elevated: 4.1 weeks *vs.* normal: 6.6 weeks, p < 0.001). The median duration of 1L therapy among patients with the normal baseline CA 19-9 levels was 11 weeks (IQR: 4-24) while the median duration was 10 weeks (IQR: 3-23) among patients with the elevated baseline CA 19-9 levels and 8 weeks (IQR: 2-20) for those who were not assessed for CA 19-9 levels (p < 0.001). However, patients with elevated CA 19-9 levels at the start of treatment had worse median OS (mOS) compared to those with normal CA 19-9 levels at baseline (**Figure 1** and **Table 3**). The mOS for the patients who received 1L, 2L, and 3L treatments with elevated baseline CA 19-9 level was lower than the mOS of the patients with the normal baseline CA 19-9 (**Table 3**).

**Table 2:** CA 19-9 testing patterns and results.

Characteristic	First Line Treated Patients, N = 6,118	Second Line Treated Patients, N = 2,402	Third Line Treated Patients, N = 790
**CA 19-9 Testing Frequency**			
No Testing	781 (13%)	273 (11%)	97 (12%)
Baseline Only	1,082 (18%)	388 (16%)	163 (21%)
One Test During 1L	896 (15%)	418 (17%)	148 (19%)
Multiple 1L Tests	3,359 (55%)	1,323 (55%)	382 (48%)
**Baseline CA 19-9 result**			
Normal	701 (11%)	293 (12%)	94 (12%)
Elevated	3,867 (63%)	1,683 (70%)	569 (72%)
Missing	1,550 (25%)	426 (18%)	127 (16%)
**CA 19-9 Baseline Value (U/mL)**			
Mean [SD]	19,054 [80,993]	10,546 [43,473]	13,468 [39,493]
Median [IQR]	929 [106 - 6,271]	886 [132 - 4,309]	1,346 [148 - 7,386]
Unknown	1,550	426	127
**CA 19-9 Trend during treatment**			
Decreasing/Same	2,566 (42%)	924 (38%)	252 (32%)
Increasing	920 (15%)	664 (28%)	248 (31%)
Missing/No Tests	2,632 (43%)	814 (34%)	290 (37%)
**CA 19-9 Change, Baseline to Nadir (U/mL)**			
Mean [SD]	-6,831 [77,786]	-1,649 [32,215]	2,802 [36,261]
Median [IQR]	-111 [-1,892 - 2]	-12 [-656 - 236]	0 [-376 - 866]
Unknown	2,632	814	290
**CA 19-9 Change, Baseline to Nadir (%)**			
Mean [SD]	53 [2,710]	183 [5,413]	128 [914]
Median [IQR]	-47 [-85 - 5]	-13 [-61 - 47]	0 [-44 - 62]
Unknown	2,632	814	290
**Time between CA 19-9 Tests, weeks**			
Mean [SD]	4.9 [6.0]	4.1 [4.2]	8 [11]
Median [IQR]	3.5 [2.1 - 5.6]	3.1 [2.0 - 5.0]	4 [2 - 9]
Unknown	1,863	661	260

SD, Standard Deviation; IQR, Interquartile range.

**Table 3:** Overall survival by CA 19-9 baseline value, testing frequency, and trends.

Baseline CA 19-9 level*	1L Median Overall Survival, months (95% CI)	2L Median Overall Survival, months (95% CI)	3L Median Overall Survival, months (95% CI)
**Overall**	7.2 (6.9, 7.5)	5.4 (5.2, 5.8)	4.3 (3.9, 4.7)
Normal	8.8 (7.9, 10)	7.2 (6.1, 9.2)	6.1 (5.4, 9.1)
Elevated	7.2 (6.8, 7.5)	5.2 (4.9, 5.6)	3.9 (3.4, 4.3)
Missing	6.3 (5.7, 6.8)	5.5 (4.8, 6.5)	4.3 (3.5, 5.5)
CA 19-9 Testing Status*			
No Testing	3.8 (3.4, 4.4)	3.7 (3.3, 4.8)	3.4 (2.8, 4.5)
Baseline Only	1.9 (1.7, 2.0)	1.9 (1.6, 2.2)	1.9 (1.5, 1.9)
One Test During Treatment	4.2 (3.9, 4.5)	3.3 (3.0, 3.7)	2.8 (2.4, 3.6)
Multiple Tests During Treatment	10.6 (10.2, 10.9)	7.8 (7.3, 8.2)	6.5 (5.7, 7.4)
Change in CA 19-9 from baseline*			
Decreasing/Stable	10.9 (10.5, 11.3)	8.2 (7.7, 8.5)	7.5 (6.6, 9.2)
Increasing	5.4 (5.1, 5.9)	4.3 (4.1, 4.7)	3.7 (3.4, 4.3)
Missing/No Tests	3.9 (3.6, 4.2)	3.3 (3.0, 3.8)	2.5 (2.0, 3.2)

*p-value < 0.001 for each line of therapy based on the log-rank test.

Patients who had baseline CA 19-9 assessments were more likely to have multiple CA 19-9 evaluations throughout the course of treatment, and these patients were also more likely to have elevated CA 19-9 levels. The majority (62.9%) of patients who were assessed for CA 19-9 prior to or during 1L treatment received multiple evaluations.

**Figure 2:**
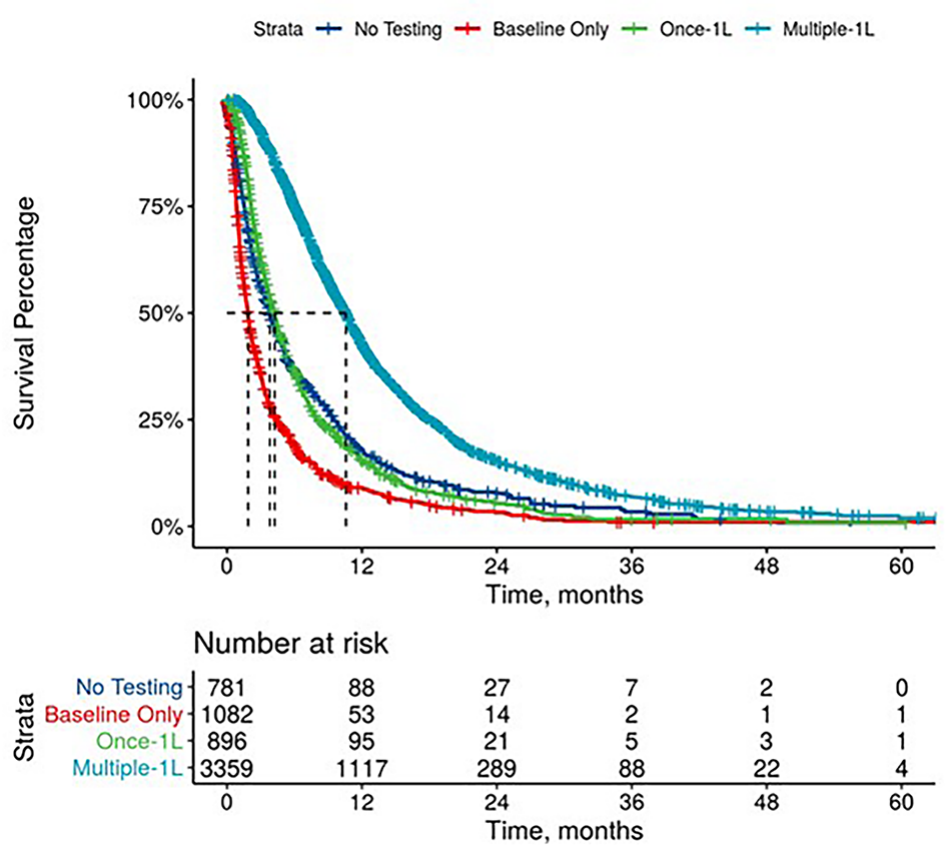
Overall Survival by Carbohydrate Antigen 19-9 Testing Status among patients treated in first line.

**Figure 3:**
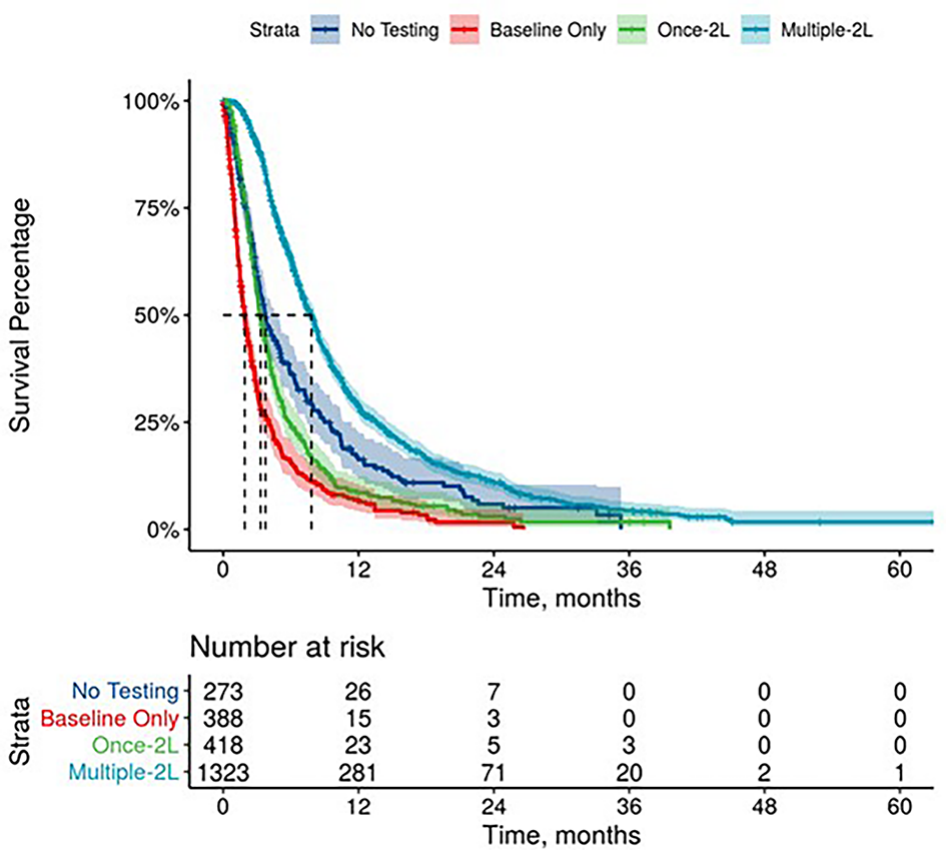
Overall Survival by Carbohydrate Antigen 19-9 Testing Status among patients treated in second line.

**Figure 4:**
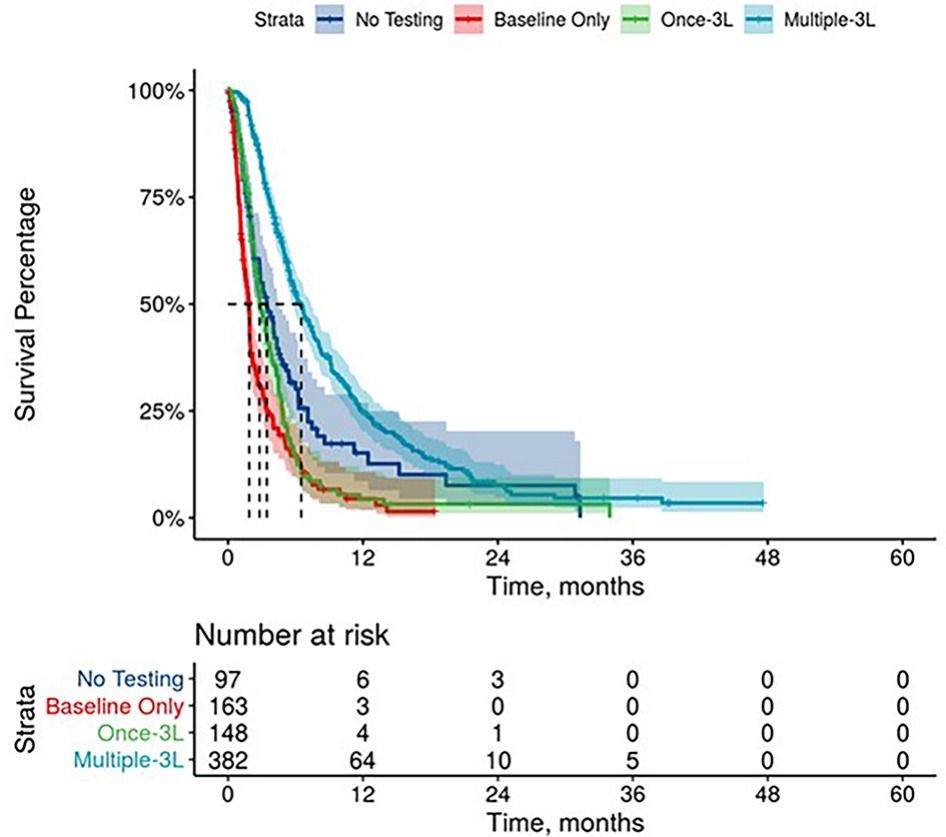
Overall Survival by Carbohydrate Antigen 19-9 Testing Status among patients treated in third line.

Further, mPDAC patients with multiple CA 19-9 tests were more likely to have better performance status and longer mOS (**Figures 2**–**4**). Patients who received multiple CA 19-9 assessments during their treatment course had the lowest proportion of patients with ECOG PS scores of 2+. Patients with multiple tests during their treatment had longer mOS than those with only 1 test or a test that only occurred prior to treatment (**Table 3**).

1L mPDAC treatment appeared to be associated with stable or decreasing CA 19-9 levels. Among the patients who were evaluable for CA 19-9 change during the 1L treatment (n=3,486), 73.6% had decreasing/the same CA 19-9 levels with a median change in CA 19-9 level of 70% (563 U/mL) while only 26.4% had an increase in CA 19-9 levels with a median change in CA 19-9 level of 56% (359 U/mL). Patients with stable and decreased CA 19-9 during 1L treatment relative to baseline had better mOS than patients whose CA 19-9 increased during treatment (**Table 3** and **Supplementary Figure 2**). Increasing CA 19-9 level from the baseline was associated with shorter median OS, and these trends remained when stratified by lines of therapy (**Table 3** and **Supplementary Figures 3**, **4**). When stratified by tertile of CA 19-9 decrease, patients treated in 1L with the largest decrease experienced the longest mOS (**Supplementary Figure 5**).

## Discussion

The results from this large retrospective observational study suggest serum CA 19-9 may serve as a prognostic tool to aid in decision-making for patients with mPDAC. An elevated CA 19-9 level at the start of treatment was associated with worse survival regardless of the line of therapy. Increasing CA 19-9 levels during the treatment relative to baseline was associated with shorter survival as well. This analysis suggests that in patients with mPDAC, both elevated and increasing CA 19-9 levels predict worse survival outcomes.

The clinical findings of this population-based study in the real-world setting are consistent with published data from prospective clinical trials (5, 17–20). The MPACT trial (NCT00844649) and the ACCORD11/PRODIGE4 trial (NCT00112658) were phase 3 randomized controlled trials (RCTs) which also investigated the role of CA 19-9 as a biomarker for mPDAC patients treated with chemotherapy in 1L settings (17, 18). The MPACT trial reported that any decline in CA 19-9 at 8-weeks from the baseline served as an early marker for chemotherapy efficacy. Additionally, the study noted CA 19-9 was more useful than radiologic assessments in identifying patients with a survival benefit. Likewise, ACCORD11/PRODIGE4 trial reported that a greater than 20% reduction in CA 19-9 at week-8 from the baseline was a predictor of significantly improved OS. The current analysis suggests that, in patients with mPDAC, stable and decreasing CA 19-9 levels (median reduction was 70%) was associated with improved median OS. A retrospective review of 8 clinical trials between April 1997 and May 2016 by Reni et al. reported that a more robust CA 19-9 response during chemotherapy (nadir), was associated with better outcome (19). mPDAC patients with CA 19-9 reduction by <50%, 50–89%, or >89% had a median survival of 7.4, 9.8, and 14.7 months, respectively (p ≤ 0.001). mPDAC patients in our study experienced a median reduction in CA 19-9 of 70% during 1L treatment and their mOS from the start of 1L was 10.9 months (IQR: 10.5-11.3). Compared to the range of OS by Reni and et al., the OS in our analysis appears to be consistent. Reni et al. also reported that the basal CA 19-9 level and the time to CA 19-9 nadir were independent predictors of OS whereas CA 19-9 reduction was not. In our study, time to CA 19-9 reduction was not measured. However, both non-elevated basal CA 19-9 levels and CA 19-9 reduction during the treatment were associated with the survival benefit. An analysis of 181 prospectively enrolled patients at a single center by Pelzer et al. reported that increased CA 19-9 was associated with a lower survival rate indicating treatment failure (20). Our analysis also reports that increasing CA 19-9 level from the baseline was associated with shorter median OS regardless of the line of therapy.

Although our study did not correlate CA 19-9 response to radiographic response, it has been reported CA 19-9 can serve as the prognostic marker in predicting tumor growth rate (5). A CA 19-9 reduction of 20% or more from the baseline has been associated with improved survival as well as significant tumor shrinkage of -0.4% per day (21).

In the past decade, a plethora of biomarkers have been evaluated to assess their predictive and prognostic utility in mPDAC management; however, CA 19-9 remains the most clinically useful and investigated biomarker for PDAC (21). Clinical assessment of treatment response in patients with mPDAC may be confounded early in their treatment course due to the difficulty in separating disease related symptoms from treatment related toxicity. A robust biomarker like CA 19-9 can help differentiate the responders from non-responders before radiographic response assessment, thus maximizing treatment benefit for the responders and minimizing toxicity for the non-responders. Since the vast majority of patients with mPDAC experience a modest survival benefit with treatment, early identification of non-responders is pivotal, so that they do not lose the window for subsequent lines of therapy. Further, a down trending CA 19-9 may be an early indication for dose attenuation in a responder experiencing treatment related toxicity. Therefore, CA 19-9 monitoring should be employed early and serially in mPDAC patients with an elevated CA 19-9 level at diagnosis. Further, the prognostic value associated with CA 19-9 trends during treatment can inform providers, patients, and their families to make meaningful treatment choices while dealing with a devastating disease.

### Limitations

Our study provides important validation regarding the utility of CA 19-9 as a biomarker for the mPDAC population in real-world settings with one of the largest and most up-to-date data sources. However, some limitations inherent to retrospective observational studies are important to consider when interpreting our findings. The data collected are retrospective and collected for routine clinical care and not for research purposes. The clinical data were derived from an EHR. The recording of patient age is capped at 85 years in the database to protect patient confidentiality. The true age of some elderly patients with mPDAC and associated clinical outcomes could not be determined. In addition, these data are collected from primarily the community setting and may not be generalizable to other settings of care. Treated patients were subject to non-random allocation. The reason to forgo treatment by the patient or physician is not available in these data. Similarly, the reasons why labs were not performed for patients are unavailable. Lastly, this study did not evaluate how specific treatment regimens impacted CA 19-9 levels and further research is necessary to characterize individual treatment regimens.

## Conclusion

This study highlights the clinical utility of CA 19-9 levels and trends as a prognostic marker in patients with mPDAC. Further this study suggests the importance of serially monitoring CA 19-9 levels in patients with mPDAC to inform treatment decisions and optimize clinical outcome. Our analysis represents one of the largest contemporary real-world studies for mPDAC patients to date. In mPDAC patients with elevated CA 19-9 levels, routine serial monitoring during treatment is warranted.

## Data Availability Statement

The data analyzed in this study is subject to the following licenses/restrictions: The data that support the findings of this study have been originated by Flatiron Health, Inc. These de-identified data may be made available upon request, and are subject to a license agreement with Flatiron Health. Requests to access these datasets should be directed to DataAccess@flatiron.com.

## Author Contributions 

BG: conceptualization, methodology, visualization, and review and editing. MK: conceptualization, formal analysis, investigation, methodology, project administration, resources, visualization, and writing—review and editing. AS: conceptualization, formal analysis, investigation, methodology, project administration, resources, visualization, and writing—review and editing. NL: analysis, resources, visualization, and review and editing. PC: conceptualization, formal analysis, funding acquisition, investigation, methodology, project administration, resources, and visualization. All authors contributed to the article and approved the submitted version.

## Funding

This study was sponsored by Ipsen.

## Conflict of Interest

BG reports a consulting/advisory relationship with Ipsen. MK, AS, and NL are employees of Genesis Research which receives funding for consulting services from Ipsen. PC is an employee of and has stock in Ipsen.

This study was sponsored by Ipsen. The sponsor had the following involvement in the study: design of the study, analysis, and interpretation as well as review of the manuscript.

## Publisher’s Note

All claims expressed in this article are solely those of the authors and do not necessarily represent those of their affiliated organizations, or those of the publisher, the editors and the reviewers. Any product that may be evaluated in this article, or claim that may be made by its manufacturer, is not guaranteed or endorsed by the publisher.

## Supplementary Material

Supplementary Figure 1 Study Cohort Attrition

Supplementary Figure 2 Overall Survival by Carbohydrate Antigen 19-9 Trend among patients treated in first line

Supplementary Figure 3 Overall Survival by Carbohydrate Antigen 19-9 Trend among patients treated in second line

Supplementary Figure 4 Overall Survival by Carbohydrate Antigen 19-9 Trend among patients treated in third line

Supplementary Figure 5 Overall Survival by Carbohydrate Antigen 19-9 decrease among patients treated in first line
