# Supplementary figures and images for: The Association of Real-World CA 19-9 Level Monitoring Patterns and Clinical Outcomes Among Patients With Metastatic Pancreatic Ductal Adenocarcinoma
Source: Front Oncol. 2021 Oct 4;11:754687. doi: 10.3389/fonc.2021.754687 (PMC8522478; doi:10.3389/fonc.2021.754687)

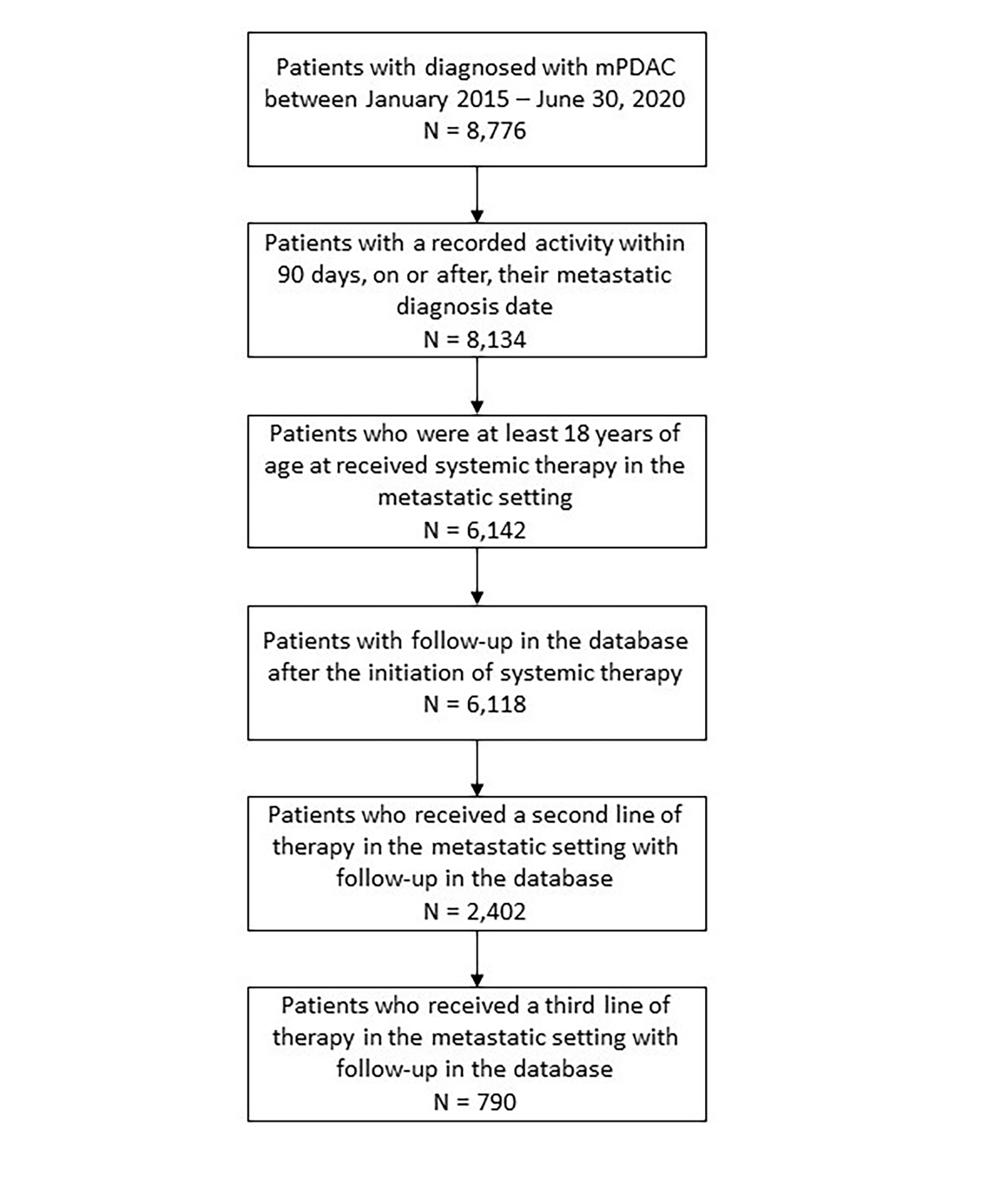

Supplement: Supplementary Figure 1 — Study Cohort Attrition [file Image_1.tif]

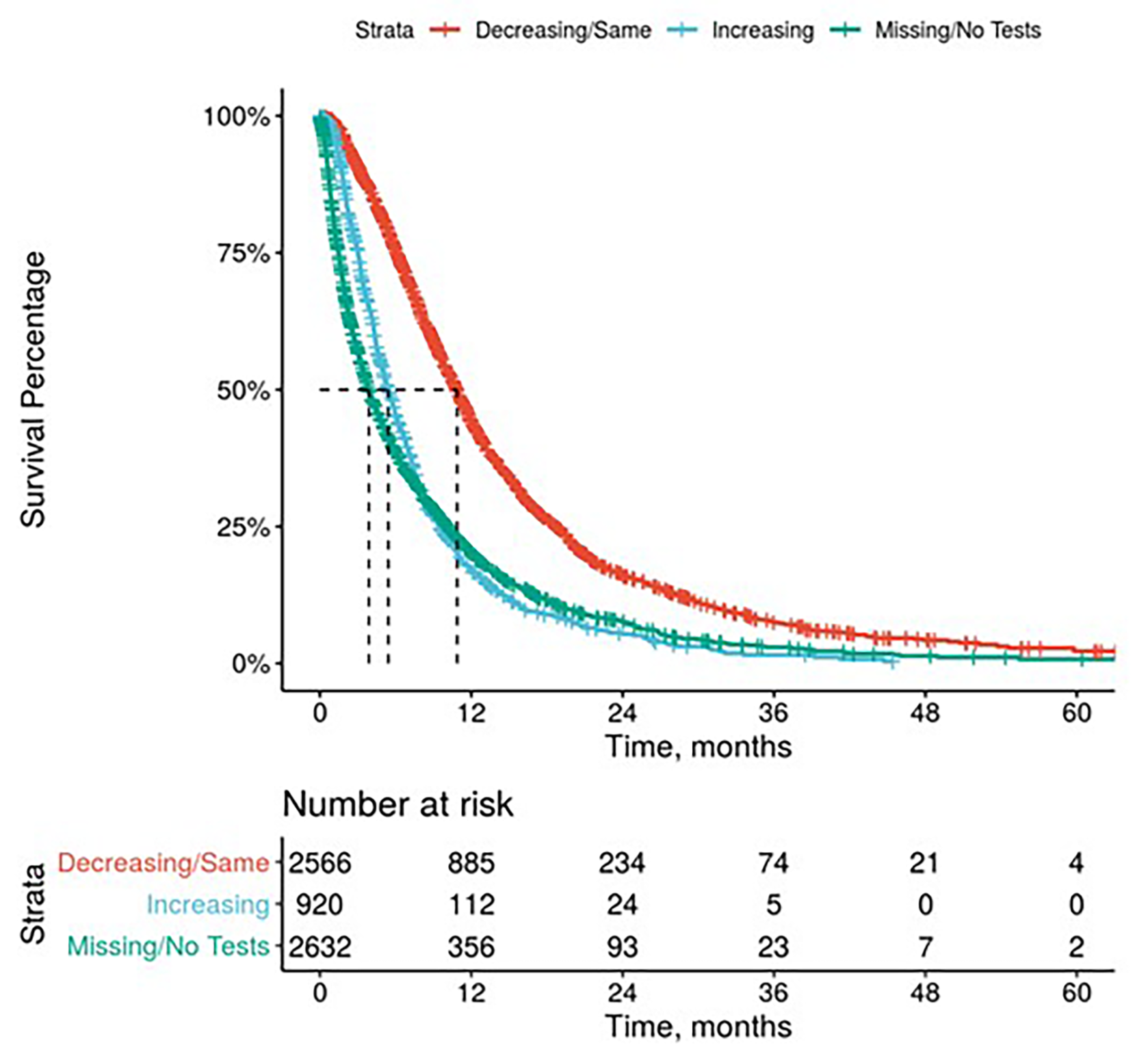

Supplement: Supplementary Figure 2 — Overall Survival by Carbohydrate Antigen 19-9 Trend among patients treated in first line [file Image_2.tif]

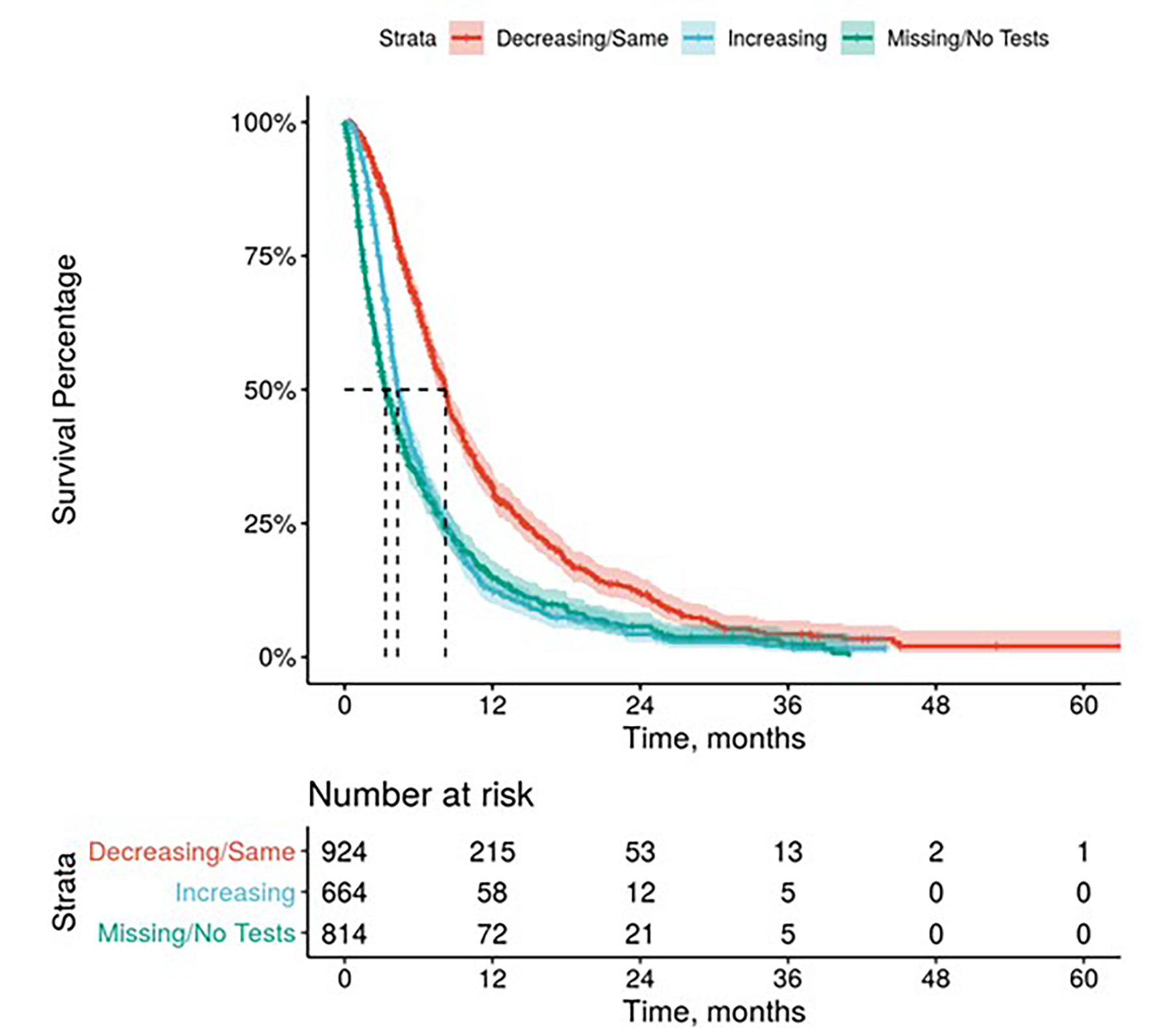

Supplement: Supplementary Figure 3 — Overall Survival by Carbohydrate Antigen 19-9 Trend among patients treated in second line [file Image_3.tif]

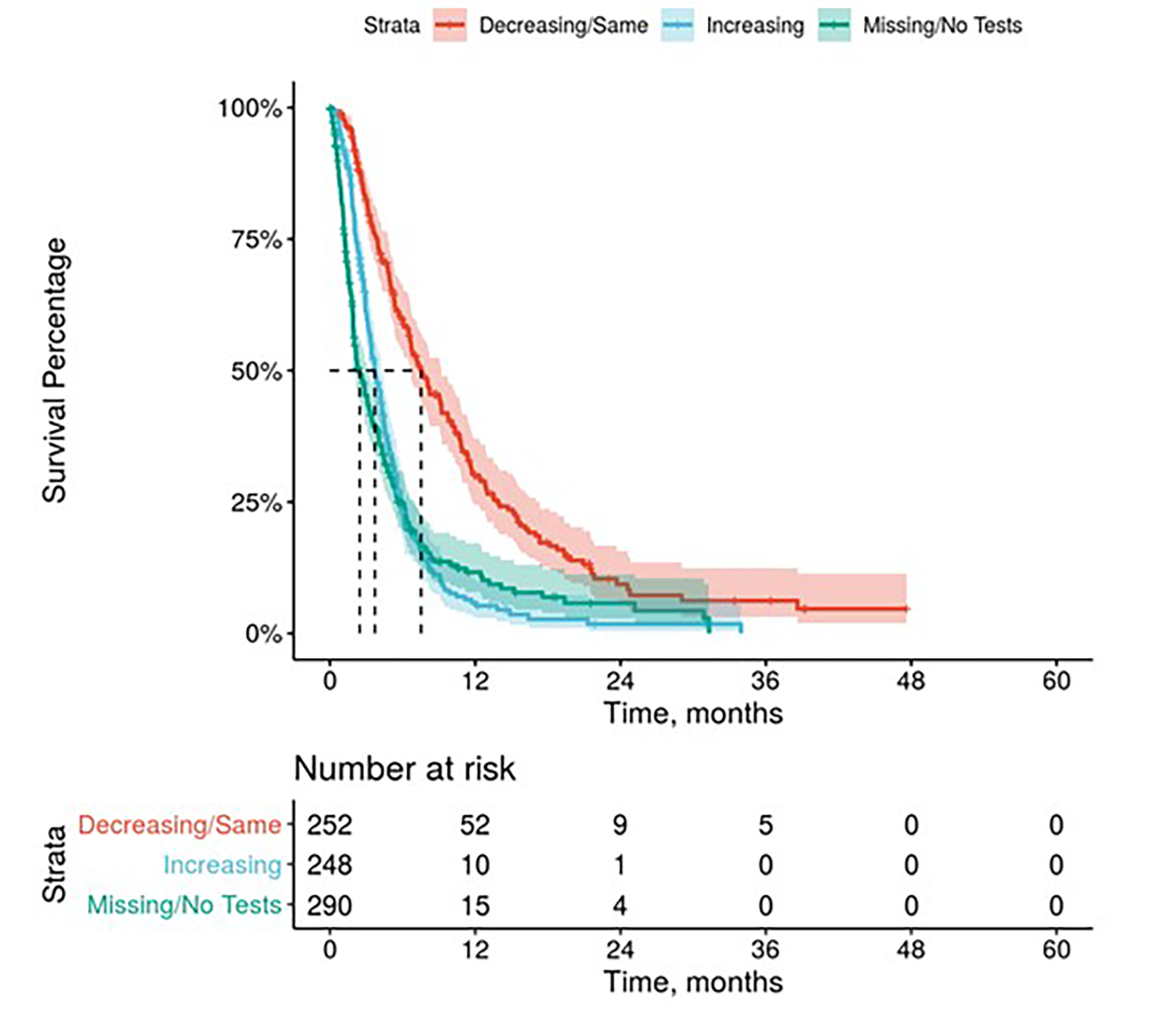

Supplement: Supplementary Figure 4 — Overall Survival by Carbohydrate Antigen 19-9 Trend among patients treated in third line [file Image_4.tif]

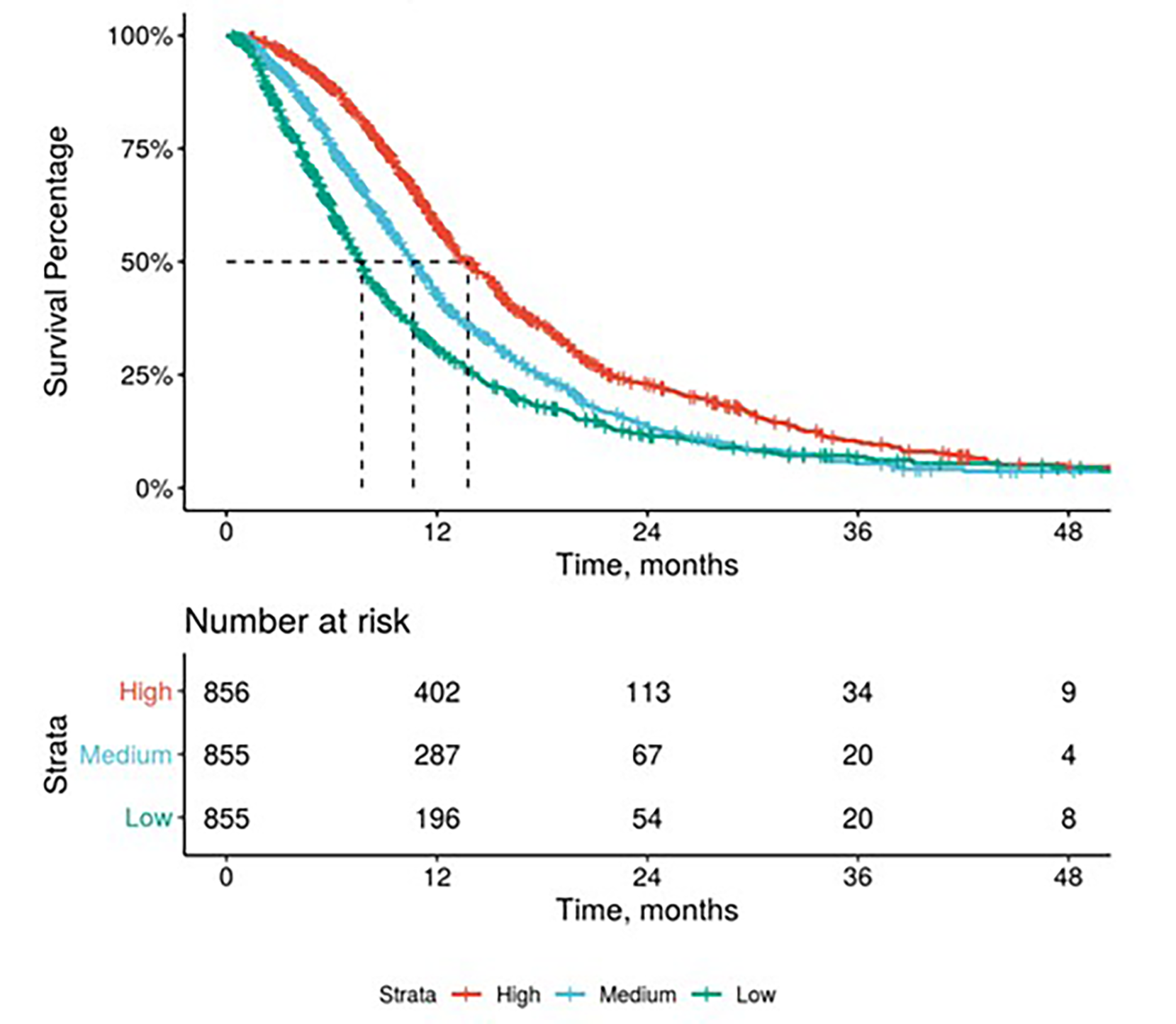

Supplement: Supplementary Figure 5 — Overall Survival by Carbohydrate Antigen 19-9 decrease among patients treated in first line [file Image_5.tif]
